# Novel Resolvin D1-Loaded Biologics as an Advanced Approach for Inflammation Control and Tissue Regeneration: Preparation and Characterization

**DOI:** 10.3390/pharmaceutics17050643

**Published:** 2025-05-13

**Authors:** Zhe Xing, Jingwen Liang, Yang Sun, Jing Dai, Jiazheng Cai, Masahito Fujio, Yiwen Xu, Xiaoli An, Ying Xue

**Affiliations:** 1 School/Hospital of Stomatology, Lanzhou University, Lanzhou 730000, China; 2 Key Laboratory of Dental Maxillofacial Reconstruction and Biological Intelligence Manufacturing, Lanzhou University, Lanzhou 730000, China; 3 Department of Medical Biology, Faculty of Health Sciences, UIT the Artic University of Norway, 9037 Tromso, Norway; 4 Zhuoruan Medical Technologies (Suzhou) Co., Ltd., Suzhou 215434, China; 5 Department of Oral and Maxillofacial Surgery, Nagoya University Graduate School of Medicine, Nagoya 464-0083, Japan; 6 Department of Clinical Dentistry, Faculty of Health Sciences, UIT the Artic University of Norway, 9037 Tromso, Norway

**Keywords:** urinary bladder matrix, biological matrix, Resolvin D1, lyophilization, inflammation resolution, osteoblastic differentiation

## Abstract

**Background/Objectives:** Constant inflammation can be a detrimental response in bone regeneration. To regulate of the inflammatory response and synchronically promote rapid tissue regeneration is a vital clinical challenge. The urinary bladder matrix (UBM) and small intestinal submucosa (SIS) composite are commonly used extracellular matrix (ECM) materials. We designed a novel drug-loaded membrane by integrating the biological matrix (BM) composed of UBM and SIS composites with Resolvin D1 (RvD1), an endogenous pro-resolving lipid mediator, using the lyophilization process. This membrane is referred to as BRL, an acronym for BM-RvD1-Lyophilization. **Methods**: In this study, the physicochemical properties of the membranes were characterized. Fluorescence staining and the CCK8 assay kit were utilized to assess biocompatibility. To evaluate the inflammatory resolution properties and osteogenic ability of osteoblasts, real-time quantitative PCR and ELISA were conducted. **Results**: BRL exhibited a more pronounced three-dimensional pore structure, demonstrating excellent physicochemical properties and enabling the slow release of RvD1. This approach improved the viability of MG63 osteoblast-like cells, reduced LPS-induced inflammation, and upregulated osteogenesis-related genes significantly. **Conclusions**: By integrating inflammation control capabilities into tissue regeneration materials, BRL effectively regulates the tissue regeneration microenvironment, thereby enhancing regeneration efficiency and positioning itself as an exceptional candidate for future tissue regeneration membranes.

## 1. Introduction

Periodontitis is an oral disease characterized by an excessive immune response that results in bone tissue damage [1]. Bone tissue engineering is considered as an ideal method for repairing alveolar bone defects, and the selection of materials is a key research topic. Most strategies in regenerative medicine and tissue engineering depend on the implantation of cells and materials to develop tissue substitutes capable of replacing damaged organs or tissues. However, implanted biomaterials inevitably induce a certain degree of inflammation in response to the presence of foreign bodies [2]. During the first week of bone tissue remodelling inflammatory factors are constantly expressed at high levels, which induces osteoclast formation through various mechanisms and influences the process of bone remodelling [3]. Therefore, research on biomaterials for bone tissue regeneration should not only focus on direct osteogenic regulation but also emphasize the reduction in local inflammatory responses.

Extracellular matrix (ECM) materials are derived from the natural tissues of organs after delipidation, decellularization, and deantigenization [4]. A large amount of decellularized tissue has been used for tissue engineering applications, including the bladder, submucosa of the small intestine, myocardium, and decalcified bone [5]. ECM materials possess the characteristics of an ideal scaffold, such as biodegradability, low immunogenicity, and the ability to provide support for cell adhesion, proliferation, migration, and differentiation [6]. Chemically crosslinked materials, commonly used in clinical settings, stimulate fibrous tissue generation by delaying material degradation or even causing chronic inflammation and residual implant material, which hinders effective tissue regeneration. The principle of tissue repair using ECM materials is “endogenous induction of regeneration”, which involves attracting and regulating host cells to grow and differentiate within the scaffold, thereby forming new tissue to replace the implanted material. Studies indicate that a key mechanism promoting endogenous tissue regeneration is the regulation of macrophage behaviour and function [7].

Urinary bladder matrix (UBM) is a unique decellularized ECM mainly composed of collagen, predominantly type I, IV, and VII collagens, along with various organic substances and growth factors [8]. It has been proved that UBM facilitates cell differentiation and tissue regeneration [9]. Due to its very low immunogenicity, UBM is an ideal material for applications such as skin replacement and wound repair, various types of hernia repairs, and dura mater repairs [10,11,12]. UBM retains the intact basement layer more effectively than other ECM materials [8] and can be synthesized into sheets-form materials to serve as a controlled and sustained drug carrier which can be precisely and rapidly placed at the site of injury [13]. Porcine small intestinal submucosa (SIS) is a widely commercialized ECM material known for its biocompatibility [14], and its fibrous and porous nature make it an excellent candidate for drug delivery [15]. The biological matrix (BM) applied in this experiment was consisted of non-crosslinked UBM (on upper and lower surfaces) and SIS (multilayers in the middle) to form a “sandwich” structure with interspaced perforations produced by vacuum compression. A similar structure has been reported to support rapid cell proliferation and migration in multiple tissue regeneration models [16].

The development of a functional BM through the combined application of biomolecules or drugs can achieve better healing quality. The UBM/SIS membrane used in this experiment (ExceFil^TM^, ZR MedTech, Suzhou, China) employed a unique non-crosslinking technology to construct a multilayer membrane structure with good mechanical properties and a low immune response. The current direction of bone tissue engineering materials has also focused on immune regulation, optimizing biomaterials to modulate inflammatory responses in the immune microenvironment and promote osseointegration between the materials and bone [17].

The resolution of inflammation is mediated by endogenous mediators, including inflammation-resolution lipid mediators such as maresins, protectins, and resolvins [18]. RvD1, a resolvin synthesized from ω-3 polyunsaturated fatty acids, has been shown to control the recruitment of neutrophils, thereby promoting the resolution of inflammation [19] without causing side effects such as gastrointestinal reactions, which are associated with the clinical use of nonsteroidal anti-inflammatory drugs [20]. In vivo and in vitro experiments have confirmed that RvD1 can inhibit macrophage pro-inflammatory polarization, promote periodontal tissue regeneration, and inhibit bone resorption caused by periodontitis [21]. Previous research indicates that the implantation of the BM into damaged tissue can lead to a significant recruitment of M2 macrophages, thereby promoting favourable tissue remodelling outcomes [22].

The goal of this study was to develop an advanced approach that combines the advantages of BM and RvD1 to simultaneously regulate inflammation and repair tissue defects. The integration is supposed to offer additional options for the surgical treatment of periodontitis and bone defects in the future.

## 2. Materials and Methods

### 2.1. Preparation of the Membranes

The BM (ExceFil^TM^, ZR MedTech, Suzhou, China) was sterilized by UV light for 24 h, lyophilized for 48 h, and then the BM of lyophilization (BL) was prepared. Under sterile conditions, RvD1 (Cayman Chemical, Ann Arbor, MI, USA) was added to the BM membrane using embedding technology. Using a micropipette, 2 mL of RvD1 solution (100 ng/mL) was carefully added dropwise to the membrane, which was then lyophilized for 48 h to prepare the lyophilized RvD1-loaded BM drug membrane (BRL). Another group was introduced with RvD1 in the same way and air-dried to make non-lyophilization RvD1-loaded BM drug membrane (BR).

### 2.2. Characterization of Membrane Properties

The surface morphology of the four membranes was examined using a scanning electron microscope (SEM, APERO-S, Washington, DC, USA). The cross-section of the membranes was observed by hematoxylin-eosin (HE) staining. A Fourier transform infrared spectroscopy instrument (FTIR, TENSOR27, Bruker, Ettlingen, Germany) was utilized to analyze the chemical composition and functional groups. The hydrophilicity and hydrophobicity of the membrane surfaces in each group were analyzed using a contact angle tester (SZ-CAMB1, Sunzeru, Shanghai, China) and 10 μL of red colour water solutions (Ponceau S red, Sigma, St. Louis, MO, USA) was dropped onto the four types of membranes to compare the diffusion property of the membranes. The pH value of the leachate was detected using a pH detector (Mettler Toledo, Shanghai, China), and deionized water was used as the control group. A universal tensile testing machine (ElectroForce 3200, TA Instruments, New Castle, DE, USA) was employed to conduct tensile tests on both wet and dry samples.

### 2.3. Degradation Property and RvD1 Release Property

The weight of each membrane was W1. After complete drying, the samples were placed in type I collagenase (BasalMedia, Shanghai, China) and placed on a constant temperature shaking table (37 °C, 100 rpm). Samples were taken at 1, 3, 5, 7, 9, 11, 13, and 14 d. After drying, the weight was W2 and the degradation rate was calculated according to the following formula: BR and BRL membranes were added to each well of a 24-well plate (n = 3). A total of 1 mL of PBS solution was added to each well. At specific time intervals (1 h, 3 h, 6 h, 12 h, 1 day, 2 days, 3 days, 5 days, and 7 days), 500 µL samples were taken from each well. The released content of RvD1 at each time point was quantitatively analyzed using an RvD1 ELISA Kit (Jiang lai, Shanghai, China).

$$Degradation\;ratio=\frac{W1-W2}{W1}\times 100\%$$

### 2.4. Evaluation of Cell Proliferation and Cytotoxicity on Membranes

The membranes of each group (BM, BL, BR, and BRL) were cut into 20 mm pieces to fit the size of 6-well plate. Sterilized membranes were laid flat on the bottom of 6-well plates and secured with a sterilized plastic ring. Cells seeded on 6-well plates without any membranes are the tissue culture plate (TCP) group. MG63 cells (CL-0157, Procell, Wuhan, China) were evenly seeded onto the surface of each plate, which were cultured for 1, 3, 5, and 7 days. A cell proliferation kit (CCK-8, Beyotime, Shanghai, China) was used to evaluate the cell proliferation. The absorbance was measured at a wavelength of 450 nm using a microplate reader (Tecan, Grödig, Austria). Cytotoxicity was assessed using a live staining kit (Solarbio, Beijing, China). The cells were stained with an AM working solution for live cell staining.

### 2.5. Inflammatory Modulation Effect

#### 2.5.1. Real-Time Quantitative PCR (RT-qPCR)

MG63 cells were evenly seeded onto the surface of TCP, BM, BL, BR, and BRL. Except for the TCP group, each group was treated with 100 ng/mL LPS (Sigma, St. Louis, MO, USA) for 24 h. Total RNA was extracted and reverse-transcribed into cDNA. RT-qPCR was used to detect each circulation threshold (Ct) of the relevant gene. The relative expression levels of *IL-6*, *IL-1β*, and *TNF-α* were evaluated (n = 3) using the 2^−ΔΔCT^ method. The primers were synthesized by Thermo Fisher and the sequences are listed in Table 1.

#### 2.5.2. Enzyme-Linked Immunosorbent Assay (ELISA)

According to the above groups, the supernatant from each group was collected. The concentrations of IL-6, IL-1β, and TNF-α in each group were determined by ELISA (Neobioscience, Guangzhou, China).

### 2.6. Osteogenic Differentiation Property Study

#### 2.6.1. Qualitative and Quantitative Detection of Alkaline Phosphatase (ALP) Activity

In LPS-induced inflammatory conditions, MG63 cells were evenly seeded onto the surface of TCP, BM, BL, BR, and BRL, which were cultured by the osteogenic induction medium for 7 days. The plates were stained with a BCIP/NBT alkaline phosphatase chromogenic reagent kit. The culture medium from each group was placed in centrifuge tubes, and the supernatant was used for this step. ALP activity was measured by an alkaline phosphatase assay kit (Nanjing Jiancheng, Nanjing, China), and the absorbance was measured at a wavelength of 450 nm using a microplate reader (Tecan, Grödig, Austria).

#### 2.6.2. RT-qPCR

In LPS-induced inflammatory conditions, MG63 cells were evenly seeded onto the surface of the membranes, which were cultured in the osteogenic induction medium for 7 and 14 days. The expression of *ALP*, *runt-related transcription factor 2* (*RUNX2*), and *osteocalcin* (*OCN*) was evaluated using RT-qPCR. The primers were synthesized by Thermo Fisher (Waltham, MA, USA) and the sequences are listed in Table 2.

### 2.7. Statistical Analysis

All experiments were repeated at least three times and all measurements were expressed as mean ± standard deviation and statistically analyzed with GraphPad Prism software (version 10.3.1). The normality of data distribution was determined using the Shapiro–Wilk test and Bartlett’s test for equal variances. A one-way ANOVA analysis was performed for statistical evaluation and differences were considered statistically significant at *p* < 0.05.

## 3. Results

### 3.1. Microstructure of the Membranes

**Figure 1:**
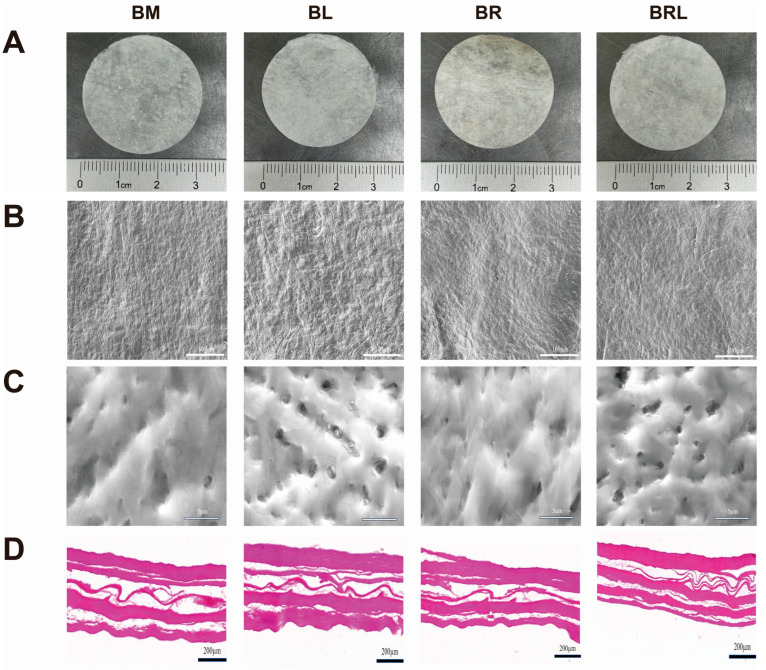
(**A**) Appearance of the membranes; (**B**) SEM images of the membranes, scale bar = 100 µm; (**C**) SEM images of the membranes, scale bar = 5 µm; (**D**) HE staining of the membranes, scale bar = 200 µm.

The BM and BL membranes are both white, opaque, and soft in texture and the BR and BRL membranes are light yellow, opaque, and can be processed into various shapes (Figure 1A). The surface features of the four membranes were observed using a scanning electron microscope. At low magnification, it was observed that each group of membranes was composed of a network of fibre structures with a relatively flat surface (Figure 1B). At high magnification, it was observed that the BL and BRL membranes were more porous and had a three-dimensional pore structure (Figure 1C). HE staining results are shown in Figure 1D and they show that all groups exhibited a uniform matrix and filamentous collagen fibres, with no residual blue-purple cell nuclei.

### 3.2. FTIR

**Figure 2:**
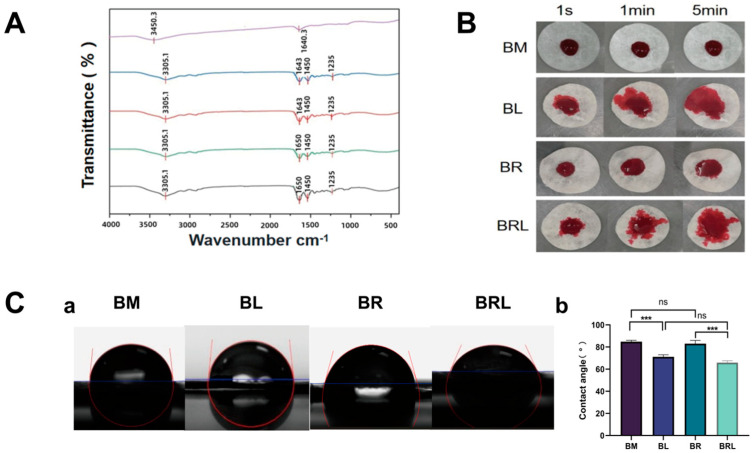
(**A**) The FTIR spectra of the membranes; (**B**) Diffusivity analysis of the membranes; (**C**) (**a**) Contact angle of the membranes; (**b**) The hydrophilicity and hydrophobicity analysis of the membranes. (*** *p <* 0.001, ns: *p* > 0.05).

In the FTIR comparison curve in Figure 2A the peak near 1640.3 cm^−1^ is due to the stretching vibration of the C=O bond and the peak at 3450.3 cm^−1^ can be attributed to the stretching vibration of intermolecular hydrogen bonds (O-H), both of which can be observed in the original RvD1 solution. The vibration peaks at approximately 3305.1 cm^−1^, 1450 cm^−1^, and 1235 cm^−1^ in the infrared spectrum of the BM membrane correspond to the stretching vibrations of N-H and the characteristic triple helical structure of collagen protein. The 1643 cm^−1^ peak on the BM and BL membranes was redshifted at 1650 cm^−1^ in the BR and BRL membranes, and the peak intensity was strongest in the BRL membrane.

### 3.3. Diffusion Property

A total of 10 μm rhodamine red solution was dropped onto the four types of membranes, and photographs were taken at 1 s, 1 min, and 5 min intervals. As shown in Figure 2B, the diffusion areas of the BL and BRL membranes were larger than those of the other two membranes.

### 3.4. Contact Angle

The contact angles for the BM, BL, BR, and BRL were 84.76 ± 1.072°, 71.16 ± 1.475°, 83.04 ± 2.359°, and 65.99 ± 1.270°. The contact angle of the BL was less than that of the BM, and the contact angle of the BRL was less than that of the BR (Figure 2C). There was no statistically significant difference in the contact angles between BM and BR.

### 3.5. PH Values

**Figure 3:**
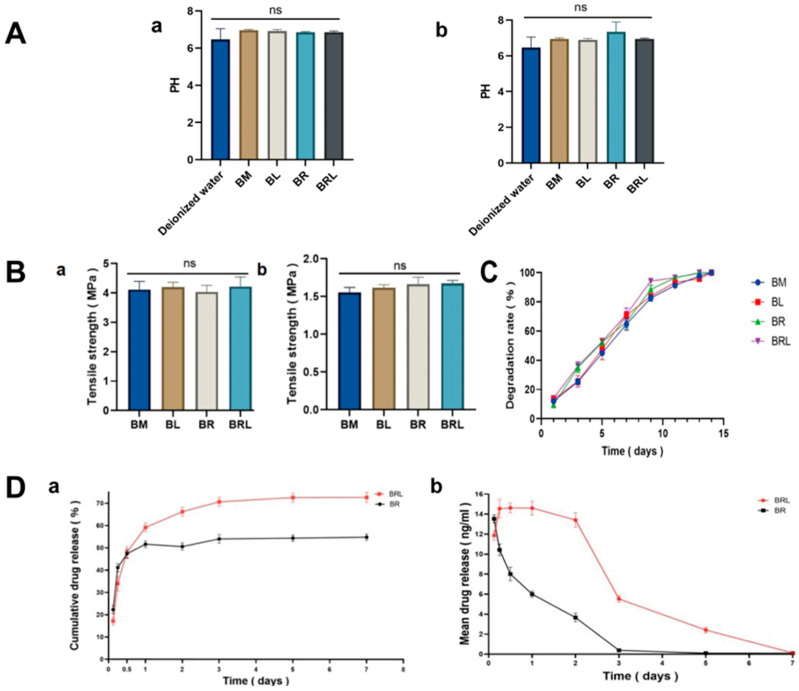
(**A**) PH of extracts from the four membranes (**a**) at 1 day and (**b**) at 7 days; (**B**) Tensile strength of the four membranes (**a**) in dry state and (**b**) in wet state; (**C**) The degradation rate of the four membranes; (**D**) Changes in (**a**) the cumulative and (**b**) the average in vitro release of RvD1 in the BR and the BRL membrane over time. (ns: *p* > 0.05).

The pH values of the leachates from the four membranes at 12 h and 24 h were neutral (Figure 3A).

### 3.6. Mechanical Property

The tensile strength of the membranes in the wet state was slightly reduced. However, there was no statistically significant difference in the tensile strength among the four groups in both dry and wet states, with all values above 1.5 MPa (Figure 3B).

### 3.7. Degradation Property

After five days of collagenase treatment, the degradation rate of the four membranes reached 50%. After 13 days, no solid matter was found in the collagenase degradation solution (Figure 3C).

### 3.8. RvD1 Release Behaviour

Both membranes released the drug rapidly in the first 12 h, followed by a gradual decrease in RvD1 release until the end of the study (7 days). Compared to the BR membrane, the BRL group exhibited a delayed release of RvD1, followed by the release of a higher concentration of RvD1 (Figure 3D). The total release of RvD1 was 53.55 ± 1.258% for BR and 73.85 ± 1.491% for BRL. In the BRL group, RvD1 was continuously released throughout the entire testing process.

### 3.9. Cytocompatibility Test

**Figure 4:**
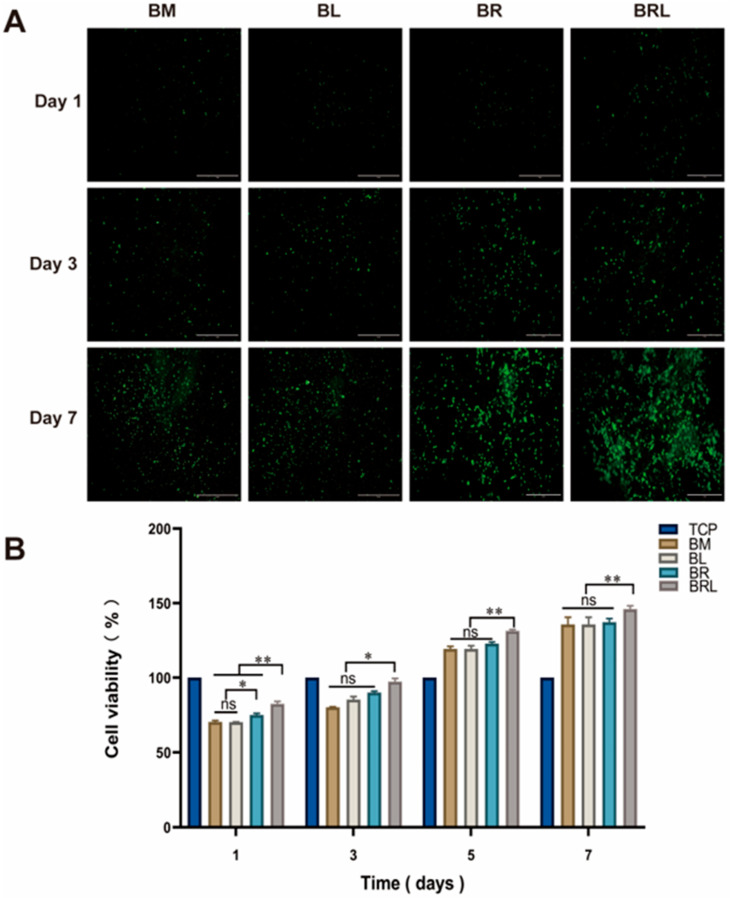
(**A**) Fluorescent staining of live cells on each group of the membranes at 1, 3, and 7 days, scale bar = 1 mm; (**B**) CCK-8 was used to detect the effects of different membranes on the activity of MG63 cells. (**: *p* < 0.01, *: *p* < 0.05, ns: *p* > 0.05).

Live cells exhibited green fluorescence in all four groups. At 7 days, it was observed that some cells were tightly connected and that the cells were clustered on the membranes and grew well (Figure 4A). As shown in Figure 4B, on the 1st, 3rd, 5th, and 7th days, the BRL group had a more significant cell proliferation effect (*p* < 0.01). There was no significant difference between the BM and BL group during the assessment period (*p* > 0.05). On the 1st day, the cell proliferation rate in the BR group was higher than that in the BM group (*p* < 0.05).

### 3.10. Analysis of Inflammatory Modulation Effect

**Figure 5:**
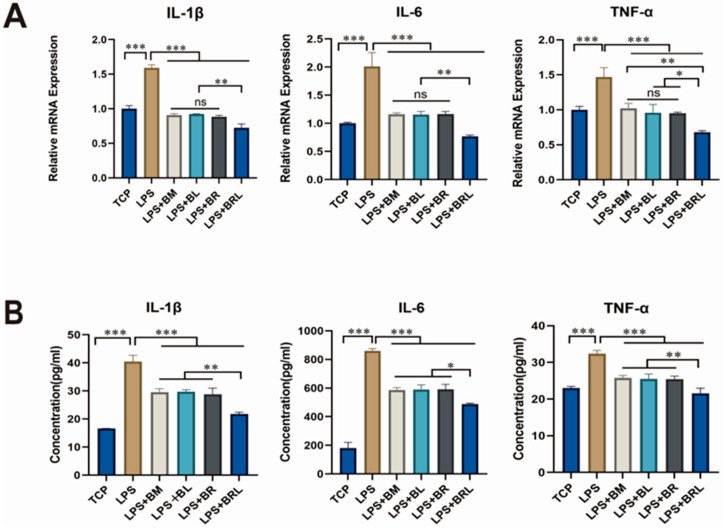
(**A**) Expression of inflammatory genes of *IL-1β*, *IL-6*, and *TNF-α* under different treatment conditions (TCP, LPS, LPS + BM, LPS + BL, LPS + BR, and LPS + BRL); (**B**) The concentrations of IL-1β, IL-6, and TNF-α in the supernatant of MG63 cells were determined under different treatment conditions (TCP, LPS, LPS + BM, LPS + BL, LPS + BR, and LPS + BRL). (***: *p* < 0.001, **: *p* < 0.01, *: *p* < 0.05, ns: *p* > 0.05).

RT-qPCR result was depicted in Figure 5A. Compared to the TCP group, the LPS group exhibited significantly increased gene expression levels of *IL-1β*, *IL-6*, and *TNF-α* (*p* < 0.001). Compared to the LPS group, the presence of membranes in all groups significantly reduced the mRNA expression of inflammatory factors *IL-1β*, *IL-6*, and *TNF-α* induced by LPS in MG63 cells (*p* < 0.001). Among all groups, the BRL group exhibited the most significant reduction in mRNA expression levels of these factors. As illustrated in Figure 5B, ELISA showed that LPS treatment elevated the secretion of IL-1β, IL-6, and TNF-α in MG63 cells (*p* < 0.001). Among the four groups, the concentrations of these inflammatory factors in the supernatant were most significantly decreased in the BRL group.

### 3.11. ALP Activity Analysis

**Figure 6:**
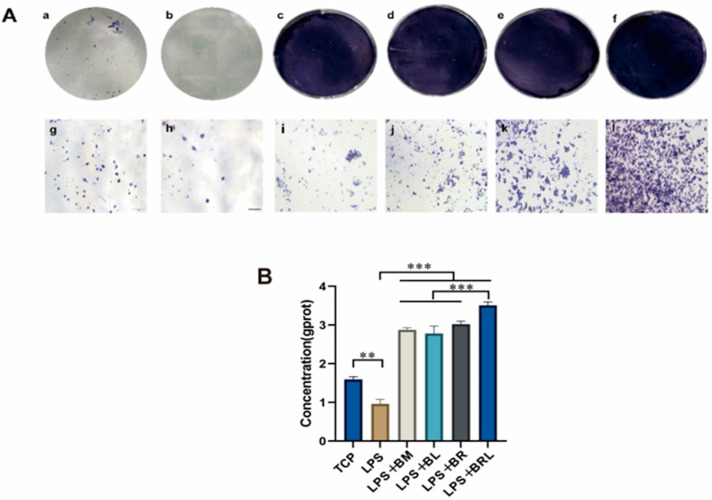
(**A**) ALP staining after 7 days of osteogenic induction under different treatment conditions: TCP (**a**,**g**), LPS (**b**,**h**), LPS + BM (**c**,**i**), LPS + BL (**d**,**j**), LPS + BR (**e**,**k**), and LPS + BRL (**f**,**l**) (×20); (**B**) Quantitative analysis of ALP staining. (***: *p* < 0.001, **: *p* < 0.01.).

As shown in Figure 6A, after 7 days a notably more intense purple colour was observed in the BRL group compared to the other groups. As shown in Figure 6B, the ALP activity in the supernatant of the LPS group was significantly lower than that of the TCP group (*p* < 0.01). Compared with the LPS group, ALP expressions in the four membrane groups were significantly elevated compared to the LPS group after 7 days (*p* < 0.001). Notably, ALP expression in the LPS + BRL group was significantly higher than in the other experimental groups *(p* < 0.001).

### 3.12. Osteogenesis-Related Gene Expression Assay

**Figure 7:**
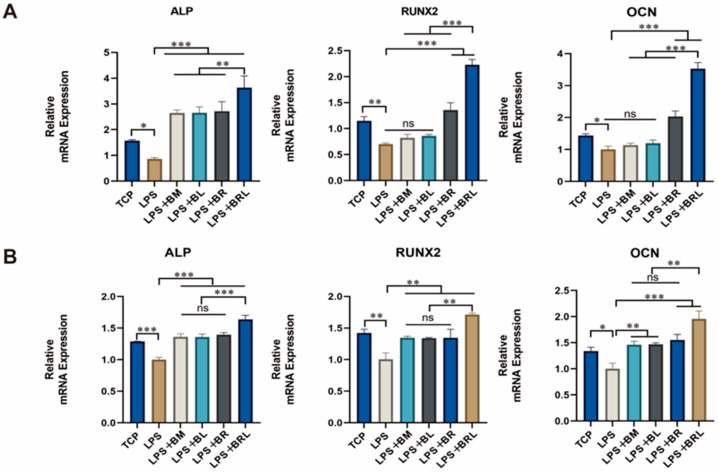
Expression of osteogenic genes of *ALP*, *RUNX2*, and *OCN* under different treatment conditions (TCP, LPS, LPS + BM, LPS + BL, LPS + BR, and LPS + BRL) after (**A**) 7 days and (**B**) 14 days. (***: *p* < 0.001, **: *p* < 0.01, *: *p* < 0.05, ns: *p* > 0.05).

As shown in Figure 7A, compared to the TCP group, the LPS group significantly downregulated mRNA expression of *ALP*, *RUNX2*, and *OCN* (*p* < 0.001). All four membrane groups exhibited a significant increase in early osteogenic gene *ALP* expression in 7 days compared to the LPS group (*p* < 0.001). For the mid–late osteogenic genes *RUNX2* and *OCN*, neither LPS + BM nor LPS + BL groups demonstrated statistically significant differences to the LPS group (*p* > 0.05). However, both LPS + BR and LPS + BRL groups exhibited markedly increased expression of *RUNX2* (*p* < 0.001) and *OCN* (*p* < 0.001) compared to LPS group. Notably, the LPS + BRL group showed significantly higher expression of *ALP* (*p* < 0.01), *RUNX2* (*p* < 0.001), and *OCN* (*p* < 0.001) when compared to the other three membrane treatment groups. As shown in Figure 7B, after 14 days of osteogenic induction in MG63 cells, the mRNA expression levels of osteogenic genes (*ALP*, *RUNX2*, and *OCN*) exhibited a significant decrease in the LPS group compared to the TCP group. In contrast, all four membrane groups demonstrated a marked increase relative to the LPS group, with the LPS + BRL group showing the most pronounced upregulation.

## 4. Discussion

Rapid resolution of inflammation and quick transition to the repair phase are crucial for tissue regeneration [23]. The unique three-dimensional structure composed of collagen fibres in the BM serves as an excellent carrier for bioactive agents, playing a significant role in regulating cell behaviour and influencing tissue-specific cell phenotypes. It has been demonstrated that a BM membrane can promote tissue repair and regeneration [8].

Although the implantation of xenogeneic decellularized matrices can provoke host immune reactions, leading to inflammation and immune responses, selecting a BM with lower immunogenicity can mitigate these issues. The BM used in this study has been shown to enhance tissue regeneration [9], and when chosen appropriately as a carrier it can reduce healing time and improve outcomes. For optimal healing, integrating BM membranes with biologics or drugs was crucial. In this research, we incorporated the anti-inflammatory molecule RvD1 into a BM membrane to create a novel drug-loaded membrane. RvD1 facilitates the transition of macrophages from the M1 phenotype, associated with inflammation, to the reparative M2 phenotype [24].

RvD1 was incorporated into the BM membrane using embedding technology, followed by lyophilization to assess its effects. Lyophilization is a widely used process in tissue engineering that can simplify and improve the efficiency of the material preparation process [25]. To evaluate the impact of lyophilization, a BR group without lyophilization was established. We observed that all the membranes showed a rapid release on the first day, likely due to the presence of a small amount of the drug on the surface of the membranes. Compared to the BR group, the BRL membrane extended the release time of RvD1, and higher concentrations of RvD1 were detected. The cumulative release of RvD1 in the BRL membrane exceeded 70%, significantly higher than that in the BR membrane. The BR membrane released RvD1 at a lower concentration, and RvD1 might have undergone rapid degradation during the release test, resulting in undetectable levels. Lyophilization is a common and repeatable procedure that enhances the long-term stability of drug formulations [26] and it has been reported as a lipid stabilizer. That might be due to the low-temperature conditions of lyophilization helping to preserve the activity of biological macromolecules and pharmaceuticals [27]. Embedding RvD1 into the BM membrane through lyophilization may address the rapid metabolism and poor stability of RvD1 [28]. Consequently, the BRL membrane forms a more effective drug-release system, which is crucial for enhancing clinical effectiveness.

Histological staining indicated the effective removal of cellular components and that adverse inflammatory reactions or immune-mediated rejection were avoided [29,30]. Contact angle and carmine red diffusion experiments demonstrated that the hydrophilic properties of the BRL membrane surface were superior to those of other groups, facilitating cell adhesion and proliferation. It was speculated that lyophilization increased the porosity of the membrane surface [31] and enhanced the hydrophilicity of the membrane surface by increasing the hydrogen bonding on the membrane surface. Tensile tests showed that the BRL membrane had good tensile strength under both dry and wet conditions. However, the impact of lyophilization on tissue mechanics remains controversial. Some studies have demonstrated that lyophilization is a physical procedure that causes minor damage to the extracellular matrix [32]. Other studies have shown that lyophilization can result in collagen rupture, leading to a significant decline in tissue mechanical properties [33]. Additionally, lyophilization can increases the porosity of decellularized corneal scaffolds [32]. Nevertheless, additional studies have indicated that the bladder acellular matrix can retain its mechanical properties after lyophilization [34]. The BRL membrane fabricated in this study exhibited outstanding mechanical properties and the addition of RvD1 and lyophilization did not compromise the original mechanical properties of the membrane, suggesting that there existed a balance between pore structure and mechanics in the prepared membrane. Under normal circumstances, the pH of the oral cavity is within the range of 6.6 to 7.1. This balance not only ensures the reproduction and growth of various oral bacterial populations but also provides defence against external stimuli to some extent [35]. The pH values of the drug-loaded membrane eluates used in this study were within the normal range. After the drug-loaded membrane was applied to the wound surface, the drug was released from the membrane to the skin surface without causing significant fluctuations in the pH value of the surrounding tissues. The redshift in the FTIR spectrum further confirmed the successful loading of RvD1 on the BM membrane through lyophilization.

The human osteoblastic osteosarcoma cell line MG63 was derived from a 14-year-old Caucasian male with left distal femoral osteosarcoma and can grow rapidly, exhibiting relatively stable phenotypes and no interspecies differences [36]. MG63 cells have been widely used in the field of tissue engineering to investigate the effects of biomaterials on cell behaviour [37] and assess the osteogenic capacity of materials [38]. Therefore, MG63 cells were selected as the research cells for this experiment to evaluate the cytocompatibility and osteogenic activity of the membranes. The SEM images showed the porous structure of the membrane, providing a suitable microenvironment for MG63 cells. In vitro proliferation experiments indicated that the presence of RvD1 and its sustained release from the BRL membrane promoted MG63 cell proliferation more than in the other groups. According to previous studies, BM membranes can promote the proliferation of BMSCs in vitro [39]. Additionally, according to the research of Xiaofeng Jiang [21], RvD1 significantly increased MG63 cell viability, which is consistent with our results. By loading RvD1 onto the BM membrane through lyophilization, the combined effect of the two significantly promoted the proliferation of MG63 cells, playing a positive role in tissue regeneration.

Inflammation plays a crucial role in bone cell regeneration and inflammatory cytokines can influence osteoclast precursor differentiation, thus regulating bone formation and resorption [40]. Previous experiments have shown that the BM membrane has a controlling effect on inflammation; for example, when the BM membrane was transplanted into the uterine horn of rats with intrauterine adhesions, BM membrane transplantation enhanced the expression of anti-inflammatory cytokines [9]. RvD1 also had an inflammatory control effect in in vivo and in vitro experiments. Benabdoun HA and colleagues induced arthritis to mice through the intraperitoneal injection of five types of type II collagen antibodies and lipopolysaccharides. The results showed that RvD1 had anti-inflammatory effects [41]. In a study by Cao [42] and others, RvD1 downregulated the levels of *IL-1β*, *IL-6*, and *TNF-α* in LPS-induced MG63 cells. The RT-qPCR results indicated that the BRL membrane showed that four membranes reduced the expression of LPS-induced inflammatory cytokines. However, the BRL drug-carrying membrane demonstrated a higher inflammatory resolution capacity. This means that the BRL membrane can maintain a more stable concentration of RvD1 and thus activate anti-inflammatory markers more effectively, creating a good microenvironment for tissue regeneration. Based on the properties of BRL, this membrane shows potential for applications in the treatment of periodontitis and other surgical interventions for inflammation-related disease. RvD1, as an inflammatory resolution molecule, regulates cytokine expression by acting on macrophages, triggering the corresponding receptors to polarize and repolarize macrophages [43], promoting the expression of M2 macrophages, and thus facilitating tissue repair and bone regeneration [44]. However, a single dose of RvD1 does not improve bone regeneration and allograft bone integration, possibly due to its relatively short-term effects, as shown in kinetic experiments which showed that RvD1 was washed away in less than 24 h [45,46]. Hence, we incorporate RvD1 into the BM membrane to prepare a multifunctional composite drug-loaded BRL membrane. In this study, after 7 days of osteogenic induction, membranes in all groups activated and expressed early osteogenic genes in MG63 cells to varying degrees. However, only the two drug-loaded groups significantly upregulated the expression of middle and late osteogenic genes, with the BRL membrane notably promoting the expression of *ALP*, *OCN*, and *RUNX2*. Following 14 days of osteogenic induction, RT-qPCR results indicated that membranes across all groups increased the expression of early, middle, and late osteogenic genes, with the BRL membrane showing the most pronounced upregulation. This phenomenon was attributed to the slow release of RvD1. The BRL prepared by lyophilization enhanced the loading capacity of RvD1 and prolonged its action time, resulting in a final drug release that was substantially higher than that of the BR group which lacked lyophilization. This finding correlated with a significant reduction in the expression of inflammatory genes in the BRL group following LPS treatment, effectively reflecting the early inflammatory control provided by the BRL membrane. These results suggest that BRL membrane accelerates the transition into the tissue regeneration phase.

Vasconcelos [47] showed that chitosan porous 3D scaffolds embedded with RvD1 improved bone healing through immune modulation. Biodegradable anti-inflammatory nano-capsules loaded with RvD1 act on a mouse femoral head lesion model, enhancing M2 macrophage polarization and stimulating bone growth in the defect area [48]. Macrophages may adapt to changes in tissue microenvironment signals by gradually transforming various functions and phenotypes. Therefore, we hypothesized that the BRL membrane may facilitate the repair of bone tissue inflammation by modulating macrophage activity, but the precise molecular mechanisms require further validation through experimental studies.

## 5. Conclusions

A multifunctional composite drug-loaded BRL membrane was successfully developed through lyophilization, which enabled a sustained drug release rate without chemical modification or additional chemical components. This approach is controllable and easily reproducible, making it suitable for a wide range of applications. The novel membrane showed positive effects on regulating the inflammatory factors, promoting osteogenesis, and the loading and releasing of RvD1. Loading RvD1 not only enhances the adhesion and proliferation of osteoblasts on the membranes but also further upregulates the expression of key osteogenic genes, thereby improving the early osteogenic differentiation capabilities of osteoblasts. Given these promising outcomes, the BRL membrane is poised to serve as an innovative and effective barrier membrane for promoting bone regeneration, potentially transforming current approaches in regenerative medicine.

## Figures and Tables

**Table 1:** PCR primers of inflammatory and osteogenic markers.

Gene	Primer Sequence (5′-3′)
*IL-1β*	F: GTGTGTGGAGAGCGTCAACC
	R: ACAGTTCCACAAAGGCATCCCAG
*IL-6*	F: CCCCTGACCCAACCACAAAT
	R: GTGCCCATGCTACATTTGCC
*TNF-α*	F: AGTAACATGGAGCTGCAGAGGATGA
	R: TGGAGACAGGGACATCAGTCG
*GAPDH*	F: ACTCCCATTCTTCCACCTTTG
	R: CCCTGTTGCTGTAGCCATATT

**Table 2:** PCR primers of osteogenic markers.

Gene	Primer Sequence (5′-3′)
*ALP*	F: ACAGCCGCCAAGAACCTCA
	R: CACTGTCTGGCACATGTTTGTCTAC
*RUNX2*	F: CACTGGCGCTGCAACAAGA
	R: CATTCCGGAGCTCAGCAGAATAA
*OCN*	F: GCAGAGTCCAGCAAAGGTGC
	R: TCAGCCAACTCGTCAGAGTC
*GAPDH*	F: ACTCCCATTCTTCCACCTTTG
	R: CCCTGTTGCTGTAGCCATATT

## Data Availability

The original contributions presented in this study are included in the article. Further inquiries can be directed to the corresponding authors.
